# Nectar trichome structure of aquatic bladderworts from the section *Utricularia* (Lentibulariaceae) with observation of flower visitors and pollinators

**DOI:** 10.1007/s00709-018-1216-2

**Published:** 2018-02-05

**Authors:** Bartosz J. Płachno, Małgorzata Stpiczyńska, Lubomír Adamec, Vitor Fernandes Oliveira Miranda, Piotr Świątek

**Affiliations:** 1 Department of Plant Cytology and Embryology, Jagiellonian University in Kraków, 9 Gronostajowa St, 30-387 Cracow, Poland; 2 Botanic Garden, Faculty of Biology, University of Warsaw, Al. Ujazdowskie 4, 00-478 Warsaw, Poland; 3 Section of Plant Ecology, Institute of Botany of the Czech Academy of Sciences, Dukelská 135, -37982 Třeboň, CZ Czech Republic; 4 Faculdade de Ciências Agrárias e Veterinárias, Jaboticabal, Departamento de Biologia Aplicada à Agropecuária, Universidade Estadual Paulista (Unesp), São Paulo, Brazil; 5 Department of Animal Histology and Embryology, University of Silesia in Katowice, 9 Bankowa St, 40-007 Katowice, Poland

**Keywords:** Bladderwort, Sect. *Utricularia*, Lentibulariaceae, Carnivorous plant, Floral micromorphology, Nectar, Spur, Pollination, Nectary ultrastructure, Entomophily, Trichomes

## Abstract

In *Utricularia*, the flower spur is a nectary and in this organ, nectar is produced and stored. This study aimed to examine the structure of the nectary trichomes in four *Utricularia* species (*Utricularia vulgaris* L., *U. australis* R.Br., *U. bremii* Heer and *U. foliosa* L.) from the generic section *Utricularia*. We have investigated whether species with different spur morphology had similar spur anatomy and nectary trichome structure. In *Utricularia* flowers, nectar is produced by spur capitate trichomes (sessile or stalked). Our results showed that regardless of the various spur morphology, trichomes have similar architecture and ultrastructure. Head cells of these trichomes are transfer cells with an eccrine nectar secretion. Examined species differed in the micromorphology of papillae in spurs. The fly *Eristalis tenax* was found to be a pollinator of *U. vulgaris*. Small Halictidae bees seem to be pollinators of *U. foliosa*.

## Introduction

In flowering plants, typical flower spurs are tubular outgrowths of perianth organs which contain nectar for pollinators. The nectary spur has been of interest to researchers for a very long time; e.g. after analysing of the spur of orchid *Angraecum sesquipedale*, Darwin (1862) proposed the coevolution of the length of a nectar spur and its pollinator’s tongue. Nectary spur characters are strongly associated with a type of pollinator, and this organ has evolved multiple times across flowering plants (Hodges 1997). After a phylogenetic analysis of the pollination evolution syndrome in *Aquilegia*, Whittall and Hodges (2007) proposed a significant evolutionary trend for increasing the nectary spur length during directional shifts to pollinators with longer tongues. According to Box et al. (2011), spurs could have evolved in angiosperms by changes in regulatory *KNOX* gene expression. Changes in spur characters are the true evolutionary innovations underlying the rapid radiation of some genera or families including Lentibulariaceae (e.g., Hodges and Arnold 1995; Hodges 1997; Puzey et al. 2012).

Most *Utricularia* species (Lentibulariaceae family) have well-developed spurs which are outgrowths of the lower corolla lip (Taylor 1989). However, in some species, the spur may be reduced in size compared to that of other corolla parts (as in *U. dunlopii*; Płachno et al. 2016) or the spur region may be only shallowly saccate as in *U. rigida* Benj. and *U. tetraloba* P.Taylor (Taylor 1989). In contrast to other *Utricularia* species, *Utricularia simmonsii* Lowrie, Cowie & Conran, which is probably the smallest species in the genus, lacks a spur entirely (Lowrie et al. 2008).

Observations of nectar production in *Utricularia* are scare. Hobbhahn et al. (2006) were the first to prove nectar production in three terrestrial *Utricularia* species belonging to the subgenus *Bivalvaria* and presented detailed data on nectar volumes and sugar concentrations. Vogel (pers. comm. in Hobbhahn et al. 2006) observed nectar in some South American *Utricularia* species. However, according to Jérémie (1989), the autogamous *Utricularia alpina* does not produce nectar. In contrast, Abrahamczyk et al. (2017) not only recorded nectar in this species but also estimated nectar sugar concentrations. Nectar was recorded in the spurs of *Utricularia reniformis* A.St.Hil. (Clivati et al. 2014; Abrahamczyk et al. 2017), *Utricularia nephrophylla* Benj. (Abrahamczyk et al. 2017) and *U. nelumbifolia* (Płachno et al. 2017b). Hobbhahn et al. (2006) pointed out that further research was needed to determine the frequency of nectar occurrence within the genus as well as the possible correlations of nectar production with the reproductive system.

This study aimed to examine the structure of the flower nectary trichomes in four *Utricularia* species from the section *Utricularia* using light microscopy and scanning and transmission electron microscopy. We investigated whether species with different spur morphology have similar spur anatomy and nectary trichome structure. To proper understanding *Utricularia* flower function in relation to its morphology and nectar secretion, additionally, we investigated flower visitors and potential pollinators.

## Material and methods

### Plant material

The species used in this study included *Utricularia vulgaris* L., *U. bremii* Heer, *U. australis* R.Br. and *U. foliosa* L*. Utricularia vulgaris* was kept in the collection of aquatic carnivorous plants in the Institute of Botany of the Czech Academy of Sciences at Třeboň, S Bohemia, Czech Republic. Cultivation conditions are detailed by Adamec and Poppinga (2016). Open flowers were observed in outdoor conditions in order to record flower visitors or pollinators during various seasons (2012, 2017). The insects were photographed using a Nikon D810 and an Olympus SP-510UZ digital camera. Trapped insects were analysed using scanning electron microscopy (SEM) to check if *U. vulgaris* pollen grains occurred on insect surfaces.

Flowers of *U. bremii* were collected from a shallow sand-pit Cep I in Suchdol nad Lužnicí, S Bohemia, Czech Republic, in 2016 and 2017. Flowers of *U. australis* were collected from populations in Silesia, Poland, in 2010 and also from the shallow sand-pit Cep I in Suchdol nad Lužnicí in 2016 and 2017. Flowers of *U. australis* originating from N.S.W, Australia, were taken from the collection of the Institute of Botany at Třeboň. *Utricularia foliosa* flowers were collected from a natural site in Mogi das Cruzes Municipality, São Paulo State, Brazil. The herbarium voucher is deposited at the JABU Herbarium (Universidade Estadual Paulista UNESP/ FCAV). Plants with flowers were observed under field conditions to record flower visitors or pollinators during all the seasons (January to December) and at least in 2 months of a season. The insects were photographed using a camera Sony Cyber-shot DSC-HX1. Trapped insects were again analysed using SEM to check whether *Utricularia* pollen grains were present.

### Floral structure and histochemical investigations

The distribution of the secretory glandular trichomes was determined by examining whole flowers (corollas) using a Nikon SZ100 stereoscopic microscope. Floral parts, namely the spurs, were examined using light microscopy (LM), scanning electron microscopy (SEM) and transmission electron microscopy (TEM) as follows. Firstly, the tissue of the spurs was examined during anthesis (in *U. vulgaris* also in young closed flowers). Pieces of the floral tissues (spurs) were excised and fixed in a mixture of 2.5% glutaraldehyde with 2.5% formaldehyde in a 0.05 M cacodylate buffer (Sigma; pH 7.2) overnight or for several days, washed three times in a 0.1 M sodium cacodylate buffer and post-fixed in a 1% osmium tetroxide solution at room temperature for 1.5 h. Dehydration using a graded ethanol series, infiltration and embedding using an epoxy embedding medium kit (Fluka) followed. Following polymerisation at 60 °C, sections were cut at 70 nm for TEM using a Leica ultracut UCT ultramicrotome, stained with uranyl acetate and lead citrate (Reynolds 1963) and examined using a Hitachi H500 transmission electron microscope at an accelerating voltage of 75 kV.

Semi-thin sections (0.9–1.0 μm thick) were prepared for LM and stained for general histology using aqueous methylene blue/azure II (MB/AII) for 1–2 min (Humphrey and Pittman 1974) and examined with an Olympus BX60 light microscope. Some material for semi-thin sections was fixed in ethanol/acetic acid (3:1 *v*/*v*). For each species (*U. vulgaris*, *U. bremii*), the 10 trichomes were measured. The mean values for trichomes of these species were given in the “Results” section.

For SEM, the representative floral parts were fixed (as above or in ethanol/acetic acid 3:1 *v*/*v*) and later dehydrated and subjected to critical-point drying using liquid CO_2_. They were then sputter-coated with gold and examined at an accelerating voltage of 20 kV using a Hitachi S-4700 scanning electron microscope (Hitachi, Tokyo, Japan), which is housed in the Institute of Geological Sciences, Jagiellonian University in Kraków).

## Results

### *Utricularia vulgaris* (Figs. 1, 2, 3, 4 and 5)

#### General flower morphology and nectary trichome structure

**Fig. 1:**
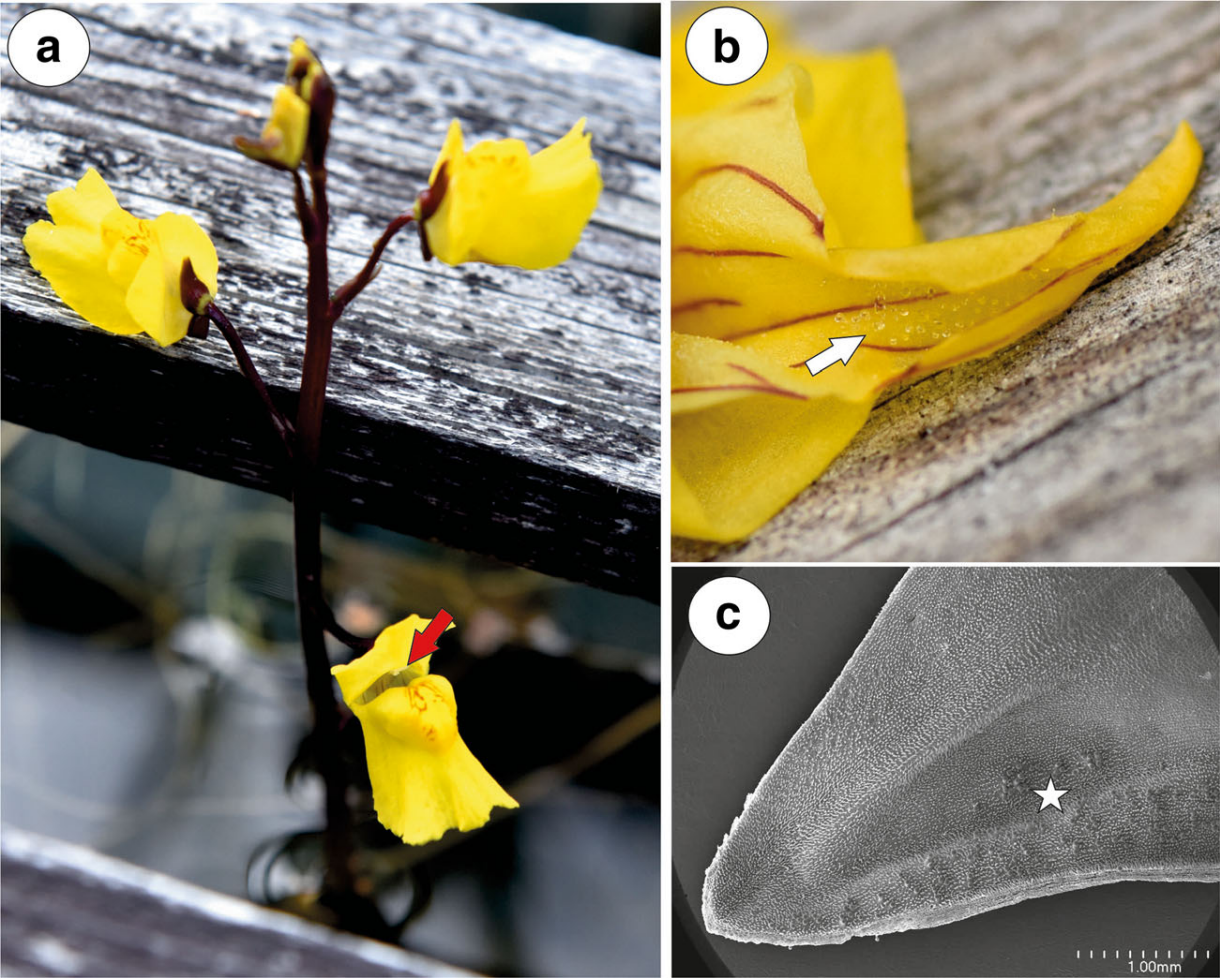
General floral morphology of *Utricularia vulgaris*. **a** Floral morphology of *U. vulgaris* from the collection of aquatic carnivorous plants in the Institute of Botany at Třeboň—visible generative structures (arrow). **b** Opened spur, nectar forms micro-droplets. **c** Section through the spur; nectar trichomes (star); bar = 1 mm

The corolla of *U. vulgaris* was yellow with reddish-brown nectar marks on the palate. The lower corolla lip formed a platform, but its margins were U-shaped deflexed. The lower lip was not firmly appressed to the upper lip, there was a slit and generative structures were visible (Fig. 1a). The spur was oriented directly downwards from the lower corolla lip at an acute angle. Nectar was observed inside the spur, forming micro-droplets (Fig. 1b). Nectar trichomes occurred on the internal abaxial side of the spur (Fig. 1c), and nectary stomata were absent.

**Fig. 2:**
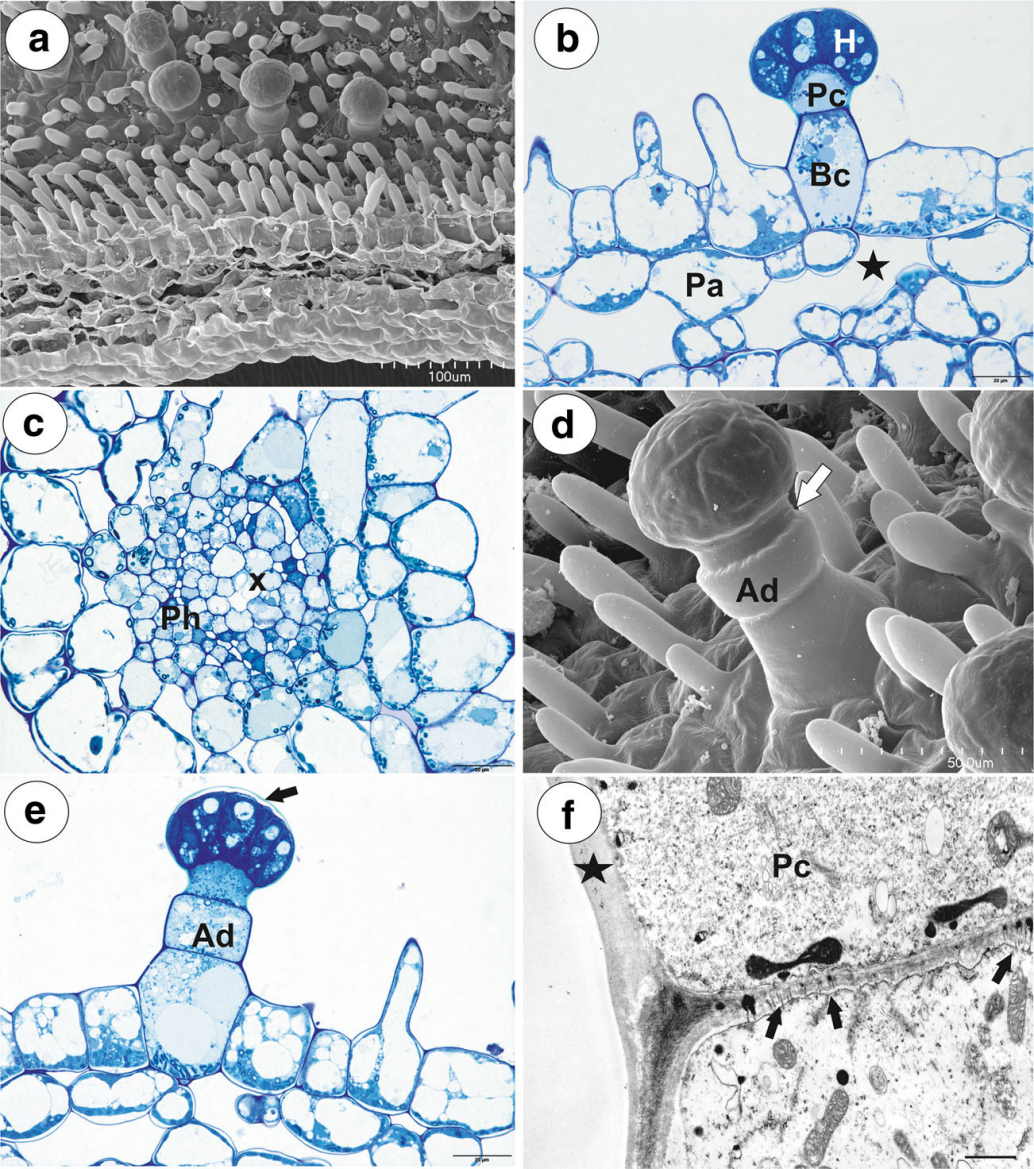
Morphology and anatomy of *Utricularia vulgaris* spur. **a** Part of the section through the spur with glandular trichomes and papillae; bar = 100 μm. **b** General structure of the glandular trichome; note that the head cells of the trichomes stain intensely with MB/AII: terminal = head cells (H), pedestal cell (Pc), basal cell (Bc), parenchyma cell (Pa), intercellular spaces (star); bar = 20 μm. **c** Part of the section through the spur showing vascular bundle: xylem elements (x), phloem (Ph); bar = 20 μm. **d**, **e** Trichomes with two-celled stalk consisting of the basal cell and the additional cell (Ad), pedestal cell (white arrow) and separated cuticle from the head cells (black arrow); bar = 50 μm (**d**) and 20 μm (**e**). **f** Part of longitudinal section through basal cell and pedestal cell (Pc), note numerous plasmodesmata (arrows) between these cells; thickened impregnated anticlinal wall of a pedestal cell (star); bar = 1.15 μm

**Fig. 3:**
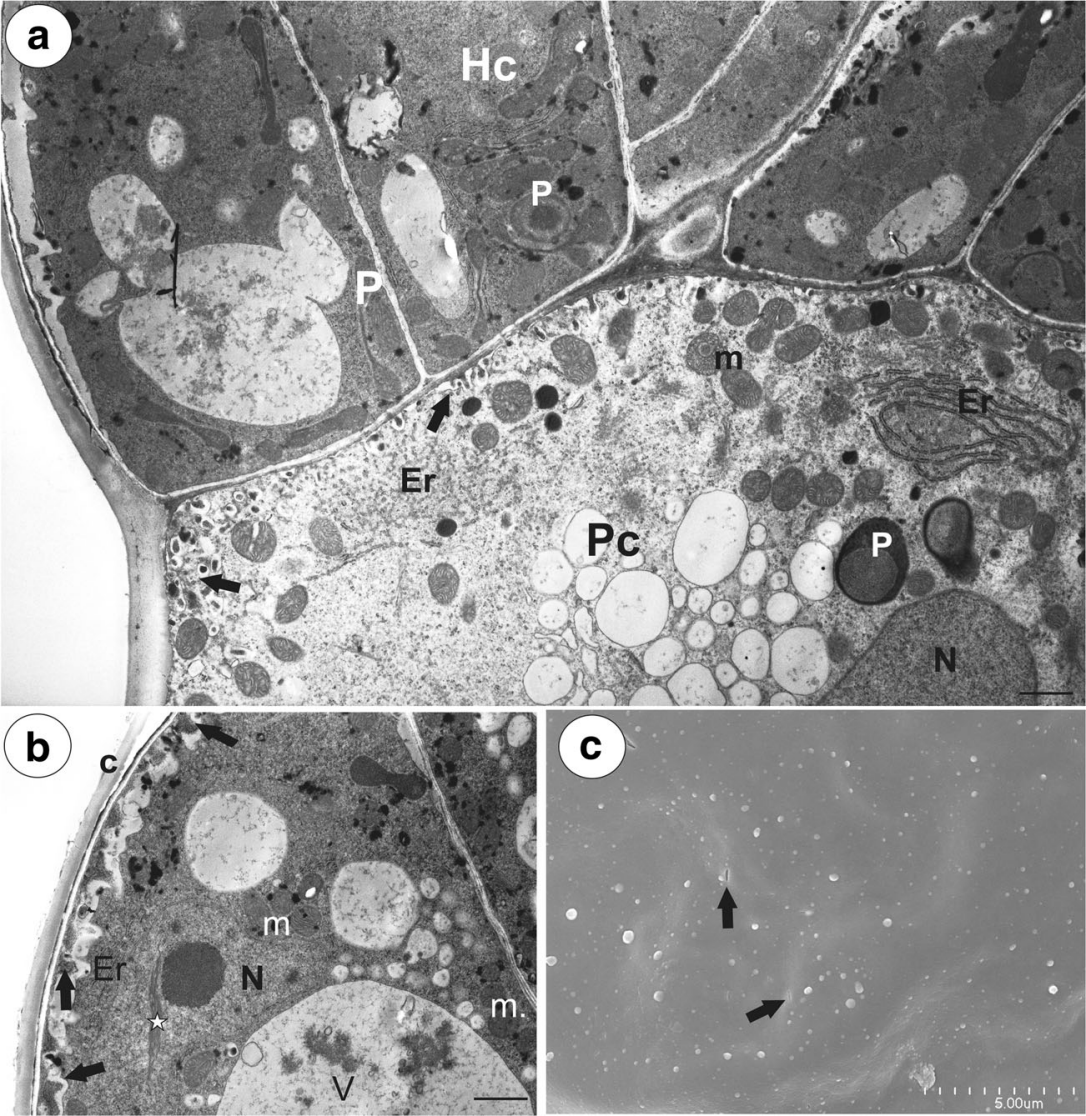
Ultrastructure of a glandular trichome from the spur of *Utricularia vulgaris*. **a** Ultrastructure of pedestal (Pc) and terminal cells (Hc); note the well-developed labyrinth wall (arrows) in the pedestal cell, plastids (P), mitochondria (m), endoplasmic reticulum (Er), nucleus (N); bar = 1.2 μm. **b** Ultrastructure of terminal cells; note the paracrystalline protein inclusion (star) in the nucleus (N), dense cytoplasm with numerous plastids, mitochondria (m). In the vacuoles (V), there is a flocculent electron-dense material. Cell-wall ingrowths (arrows) are on the inner surface of the outer wall; thick cuticle (c); bar = 1.8 μm. **c** Cuticle of head cells visible in SEM, note cuticular pores (arrows); bar = 5 μm

**Fig. 4:**
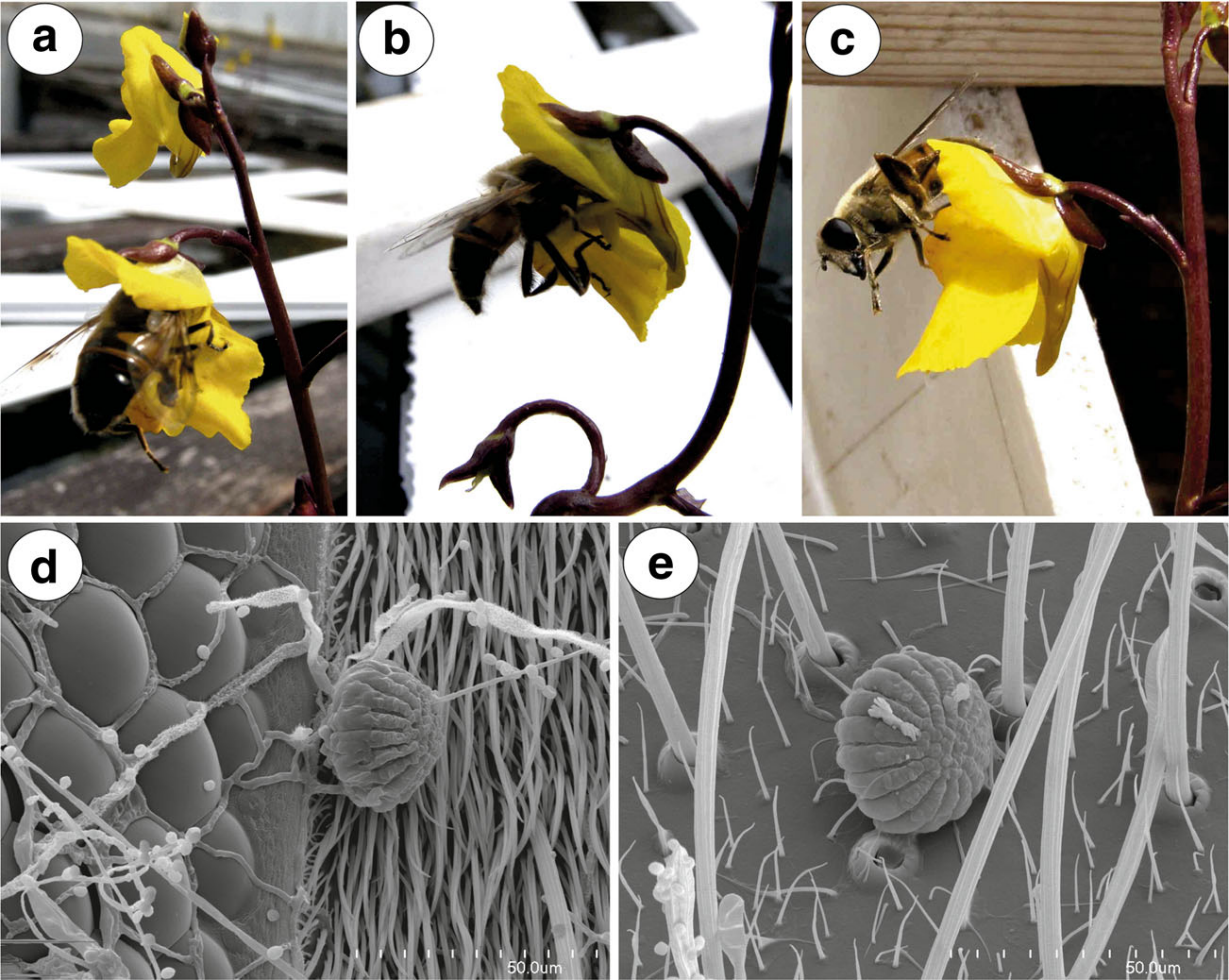
Pollination of *Utricularia vulgaris*. **a**–**c** Behaviour of *Eristalis tenax* on *U. vulgaris* flowers. **d**, **e** Pollen grains of *U. vulgaris* on the surface of *Eristalis tenax*; bar = 50 μm

**Fig. 5:**
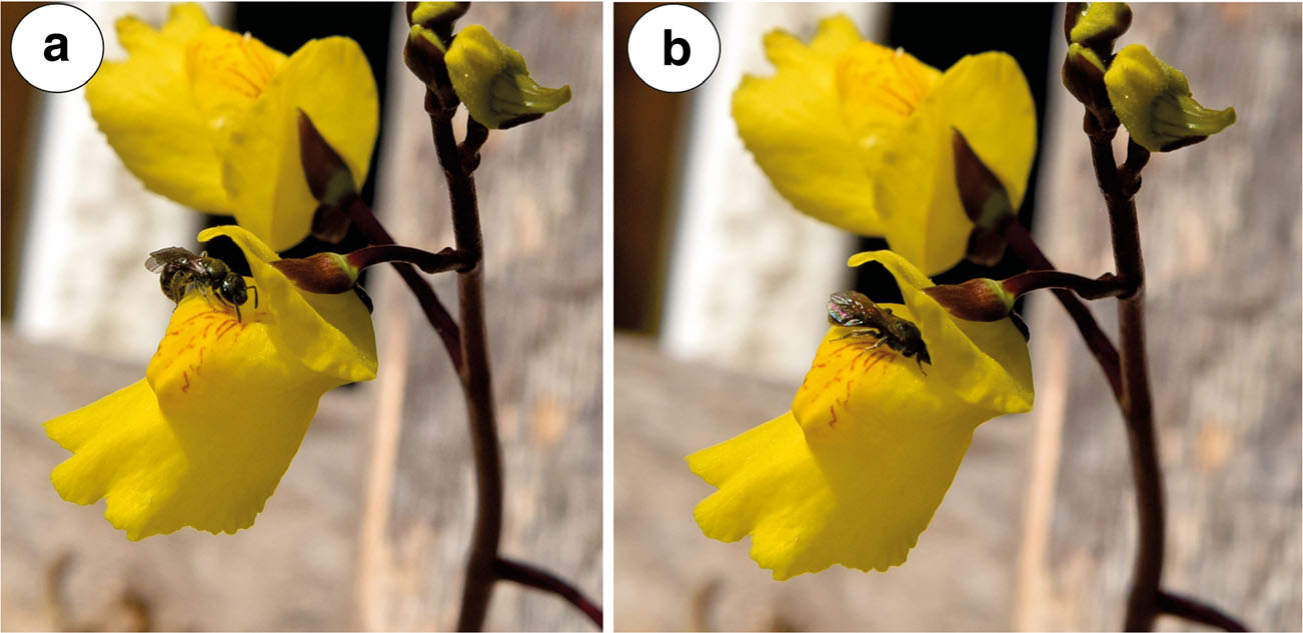
Visitors on the surface of *Utricularia vulgaris* flower. **a**, **b** Behaviour of bee *Lasioglossum* sp. on the *U. vulgaris* flower

In a transverse section, the wall of the spur was composed of several cell layers: the internal epidermis, a few layers (2–3) of parenchyma cells and the outer epidermis (Fig. 2a, b). The parenchyma cells were non-glandular. Relatively large intercellular spaces occurred between the parenchyma cells. The parenchyma cells had numerous plastids with large starch grains. Collateral vascular bundles occurred in the ground parenchyma (Fig. 2c), each containing both xylem and phloem elements. Internal epidermis formed papillae, which were unicellular with a smooth surface (Fig. 2d). They were highly vacuolated and had numerous plastids in their cytoplasm.

Glandular spur trichomes were composed of a single basal cell which formed a unicellular stalk (length 21 μm), pedestal cell (barrier cell, mean length = 17 μm, *n* = 10) (Fig. 2b) and a multi-celled head (mean length = 21 μm, *n* = 10, mean diameter = 47 μm, *n* = 10; Figs. 2b and 3a). However, part of the trichomes had a two-celled stalk, which consisted of a basal cell and an additional cell (Fig. 2d, e). The pedestal cell and the head cells were different in vacuolisation and the degree of cytoplasm density from the stalk cells (Fig. 2b, e).

The basal cell was highly vacuolated and most of the cytoplasm with the usual organelles was located in the upper part of the cell near the pedestal cell or the additional stalk cell. The nucleus of the basal cell was surrounded by numerous plastids. The lateral wall of the basal cell developed a cuticle in the place where it was not connected with other cells. Similarly, the lateral wall of the additional stalk cell developed a distinct cuticle. The additional stalk cell was more or less cylindrically shaped. Numerous plasmodesmata occurred in the transverse walls between the stalk cell and the pedestal cell as well as between the additional stalk cell and the pedestal cell (Fig. 2f). The pedestal cell had a thick radial wall, which was impregnated with cutin (Figs. 2f and 3a). Most of the cytoplasm, with the nucleus and the usual organelles (mitochondria, plastids, and endoplasmic reticulum), were located in the upper part of the cell near the head cells (Fig. 3a). Numerous plasmodesmata occurred in the transverse walls between the pedestal cell and the terminal cells. The protoplasts of the head cells were electron-dense and had a prominent nucleus containing a paracrystalline protein inclusion (Fig. 3b). The cell wall ingrowths arose from the inner surface of the outer cell walls (Fig. 3b) as well as from the inner walls between the head cells. Plastids were numerous and were dumbbell- or cube-shaped (Fig. 3a) There were also multivesicular bodies (MVBs) present. Flocculent electron-dense material was present in the vacuoles (Fig. 3b). Mitochondria with well-developed cristae and profiles of rough endoplasmic reticulum (RER) were common in the cytoplasm of the head cells. Dictyosomes were small and not hypertrophied. The thick cuticle (Fig. 3b) frequently became distended and separated from the cell walls of the head cells on the apex of the head (Fig. 2e). Small cuticle pores together with micro-droplets of secretions were observed (Fig. 3c).

#### Floral visitors and pollinators

Flowers of *Utricularia vulgaris* were visited by females of a Common Drone Fly *Eristalis tenax* (Linnaeus, 1758); family Syrphidae (Fig. 4a–c) and a small bee *Lasioglossum* sp.; family Halictidae (Fig. 5a, b). Inside the flowers, undetermined members of Thysanoptera were observed. Aphids (*Rhopalosiphum nymphaeae* L.) occurred mainly on the external parts of inflorescences, and sometimes, aphids also were seen in the flowers (not shown).

A specimen of the large fly *Eristalis tenax* used the lower lip as a landing platform and later forced open the two corolla lobes. It entered the corolla tube with the front of its body and fed on the nectar in the spur. During this process, the fly was holding its leg on the expanded-swollen part of the lower lip (limb—the term sensu Taylor 1989, a part of palate sensu us) (Fig. 4a). SEM analysis revealed that *Utricularia vulgaris* pollen grains occurred on the surface of the *Eristalis* head and thorax—both on the lateral parts as well as on the insect’s back (Fig. 4d, e). *Eristalis tenax* cleaned its surface after penetrating the flower (Fig. 4c). The *Lasioglossum* bee penetrated the palate (Fig. 5a), studied the surface of the palate using antennae (Fig. 5a) and later penetrated the corolla tube (Fig. 5b). In contrast to *Eristalis tenax*, *Lasioglossum* was not using its strength to penetrate the flower and also did not deform the corolla shape during the visit. Under SEM, we did not observe *U. vulgaris* pollen grains on the surface of the *Lasioglossum* head and thorax.

### *Utricularia australis* (Fig. 6a–d)

**Fig. 6:**
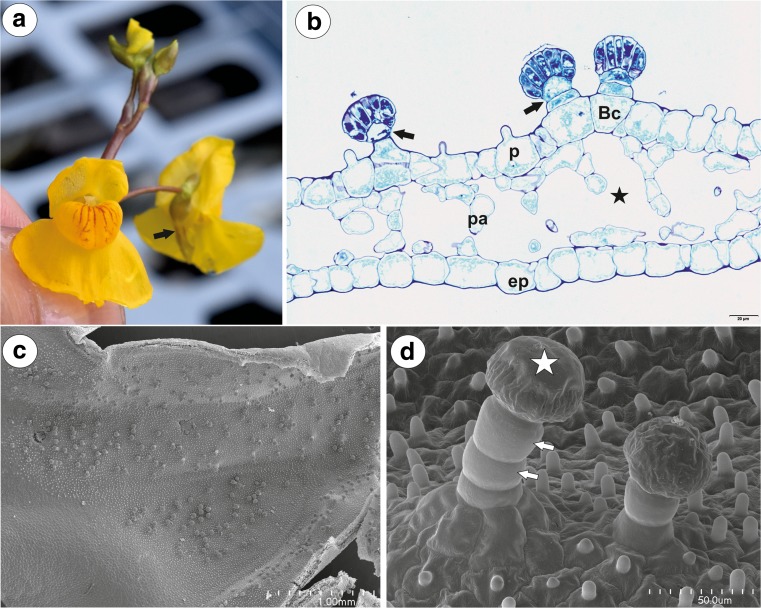
General floral morphology and spur structure of *Utricularia australis*. **a** Floral morphology of *U. australis* (Australian population) from the collection of aquatic carnivorous plants in the Institute of Botany at Třeboň: spur (arrow). **b** Part of the section through the spur with glandular trichomes and papillae: pedestal cell (arrow), basal cell (Bc), papilla (p), parenchyma cell (pa), intercellular spaces (star), external epidermis (ep); bar = 20 μm. **c** Nectar trichome distribution inside the spur; bar = 1 mm. **d** Morphology of nectar trichomes and papillae; note that one trichome had a three-celled stalk; additional cell (arrow), swollen cuticle of the head cells (star); bar = 50 μm

The corolla of *U. australis* was yellow with reddish-brown nectar marks on the prominent palate (Fig. 6a). In the transverse section, the wall of the spur was composed of several cell layers: internal epidermis, a few layers of parenchyma cells and outer epidermis (Fig. 6b). The parenchyma cells were non-glandular. Very large intercellular spaces occurred between the parenchyma cells (Fig. 6b). Two collateral vascular bundles occurred in the parenchyma, each containing both xylem and phloem elements (not shown). Nectar trichomes occurred on both the abaxial and adaxial spur side (Fig. 6c). These glandular trichomes were composed of a single basal cell which formed the unicellular stalk, the pedestal cell (barrier cell) and a multi-celled head (Fig. 6b). However, part of the trichomes had the two- or three-celled stalk, which consisted of a basal cell and one or two additional cells (Fig. 6d). Epidermal cells sometimes formed a pedestal for the trichome (Fig. 6d). The inner epidermis formed short unicellular papillae with a smooth surface (Fig. 6d). They were highly vacuolated, not glandular.

### *Utricularia bremii* (Fig. 7a–f)

**Fig. 7:**
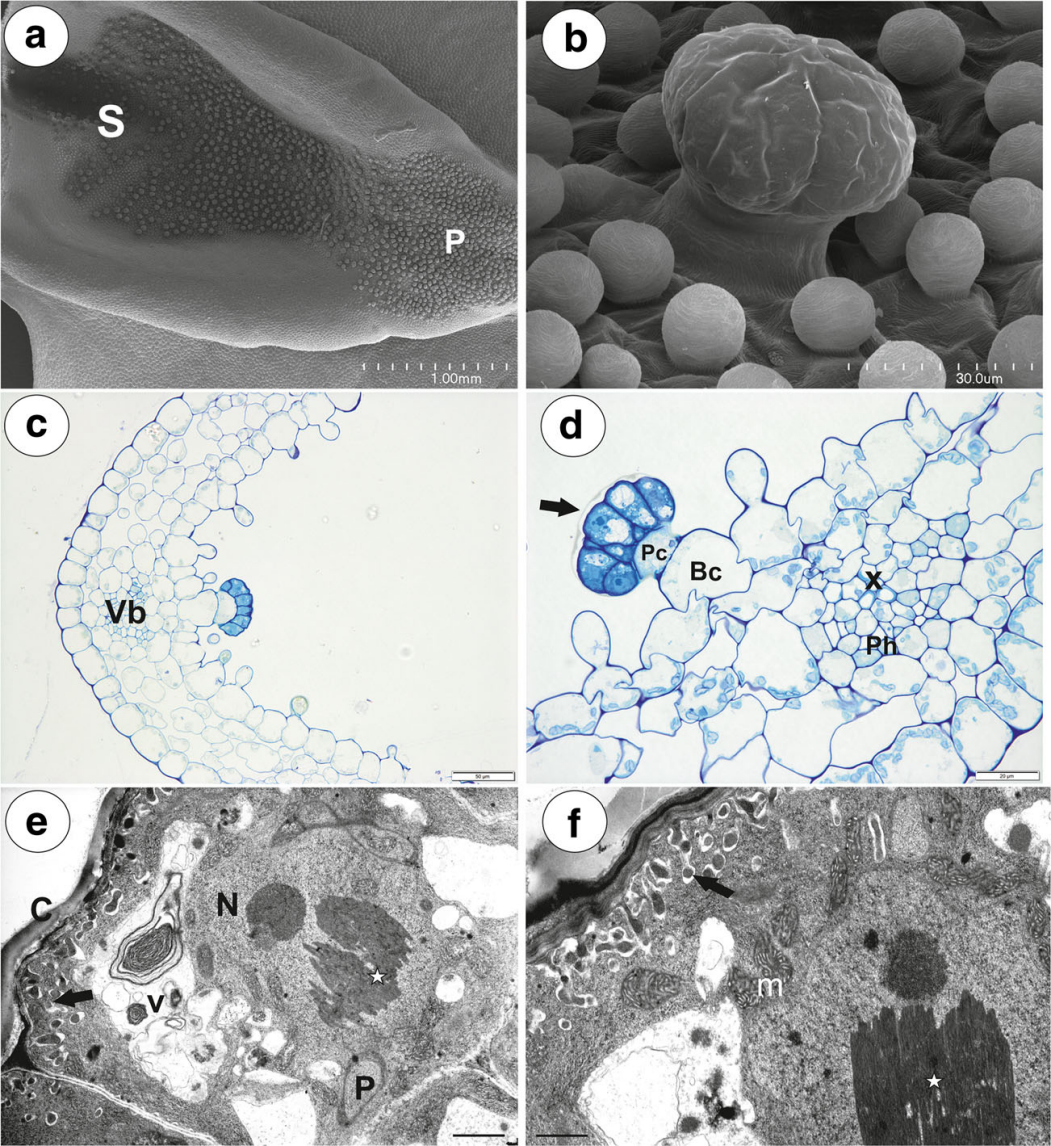
General morphology and anatomy of *Utricularia bremii* spur. **a** General morphology of the lower corolla lip: palate (P), throat spur (S); bar = 1 mm. **b** Morphology of nectar spur trichomes and papillae; bar = 30 μm. **c** General anatomy of the spur: vascular bundle (Vb); bar = 50 μm. **d** Part of the section through the spur showing structure of trichome and vascular bundle: separated cuticle from the head cells (black arrow), pedestal cell (Pc), basal cell (Bc), xylem elements (x), phloem (Ph); bar = 20 μm. **e**, **f** Ultrastructure of head cells; note the paracrystalline protein inclusion (star) in the nucleus (N), dense cytoplasm with mitochondria (m). In the vacuoles (V), there are flocculent electron-dense material and myelin-like figures. Cell-wall ingrowths (arrows) are on the inner surface of the outer wall but also on the inner walls between the terminal cells; thick cuticle (c); bar = 1.1 μm (**e**) and bar = 1 μm (**f**)

The spur was shortly conical. In the lateral view, it was about as wide as long. Glandular trichomes densely covered the internal ventral spur surface (Fig. 7a, b). There was glandular continuity between the palate and the spur (Fig. 7a). In the transverse section, the wall of the spur was composed of several cell layers: the internal epidermis, a few layers of parenchyma cells and the outer epidermis (Fig. 7c). The parenchyma cells were highly vacuolated and non-glandular. Intercellular spaces occurred between the parenchyma cells. The parenchyma cells contained numerous plastids with large starch grains. Collateral vascular bundles occurred in the ground parenchyma (Fig. 7c, d), and each contained both xylem and phloem elements. The inner epidermis formed short unicellular papillae with striations on the surface (Fig. 7b). The papillae had rounded tips. They were highly vacuolated and contained plastids with starch.

The glandular spur trichomes were composed of a single basal cell, a unicellular short pedestal cell (barrier cell with a thick radial wall impregnated with cutin, mean length = 16 μm, *n* = 10) and a multi-celled head (mean diameter = 42 μm, *n* = 10; Fig. 7d). The basal cell was highly vacuolated, and the lateral wall of this cell lay partly embedded in the epidermis. Its outer part, which formed a short “stalk”, created a well-developed cuticle that was continuous with that of the epidermal cells and the cuticular deposits of the pedestal cell. The head cells were transfer cells. Cell wall ingrowths (reticulate type) occurred on the inner surface of the outer wall of the terminal (head) cells, but also on the inner walls between the terminal cells (Fig. 7e, f). The cytoplasm of the head cells of the glandular trichomes stained deeply with methylene blue/azure II (Fig. 7d). The protoplasts of the head cells were electron-dense and had a prominent nucleus containing a paracrystalline protein inclusion. Their vacuole contained myelin-like figures and flocculent electron-dense material (Fig. 7e, f). Plastids were common and contained an electron-dense stroma; some of them were cube- or amoeboid-shaped (Fig. 7e). Mitochondria with well-developed cristae (Fig. 7f) and profiles of rough endoplasmic reticulum (RER) occurred in the cytoplasm. The thick cuticle frequently became distended and separated from the cell walls of the head cells, especially in the apical part of the cells in response to the subcuticular accumulation of the nectar.

### *Utricularia foliosa* (Figs. 8 and 9)

**Fig. 8:**
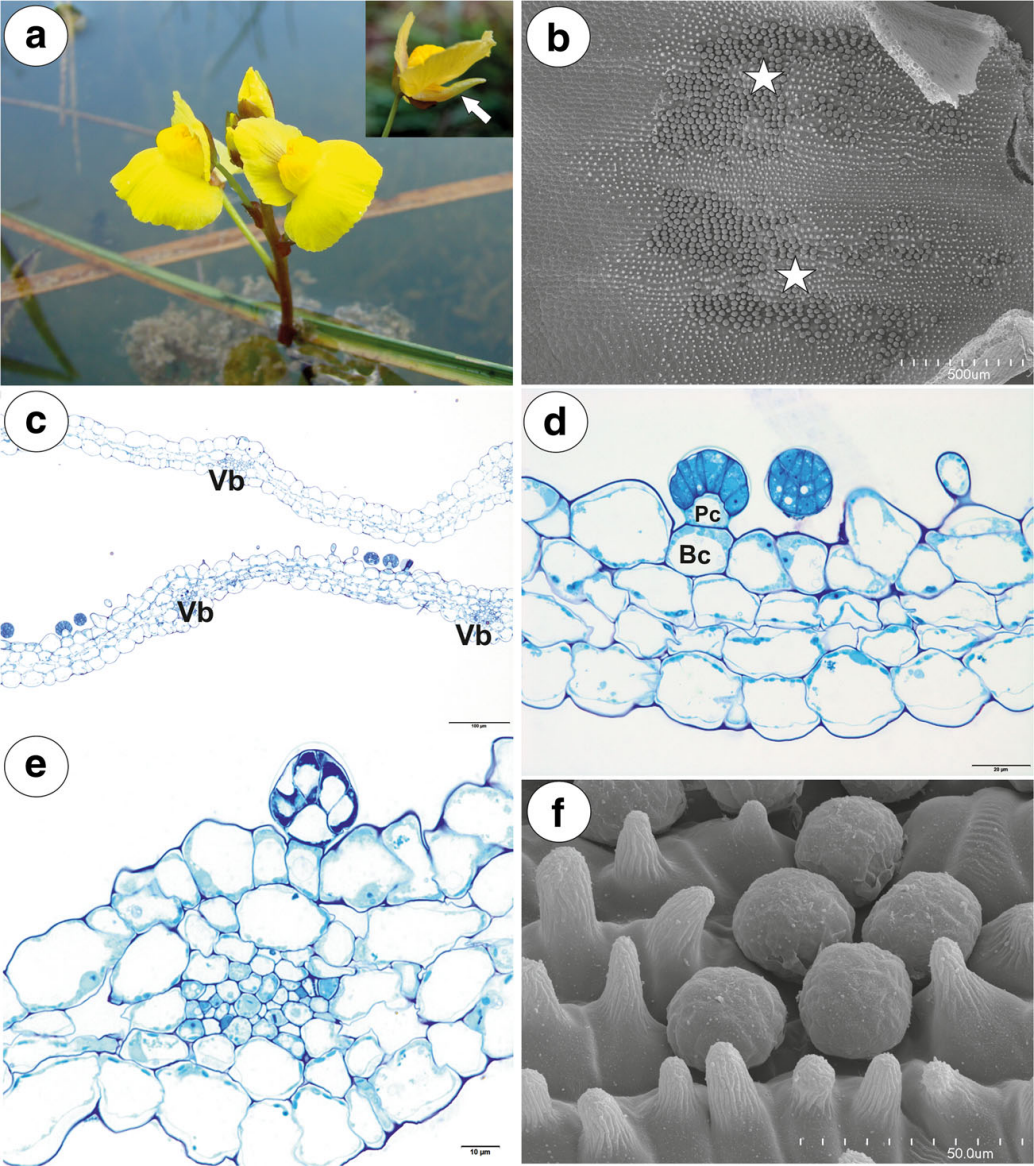
General floral morphology and spur structure of *Utricularia foliosa*. **a** Floral morphology of *U. foliosa*: spur (arrow). **b** Nectar trichome distribution inside the spur; bar = 500 μm. **c** General anatomy of the spur: vascular bundle (Vb); bar = 100 μm. **d** Part of the section through the spur showing structure of trichome: pedestal cell (Pc), basal cell (Bc); bar = 20 μm. **e** Occurrence of a nectar trichome near the vascular bundle; bar = 10 μm. **f** Morphology of nectar trichomes and papillae; bar = 50 μm

The spur was narrowly conical, nearly parallel with the lower lip (Fig. 8a). Nectariferous trichomes occurred mainly in two patches (Fig. 8b). Trichomes were also rarely scattered on the opposite spur surface. In the transverse section, the spur wall was composed of several cell layers: the internal epidermis, a few layers of parenchyma cells and the outer epidermis (Fig. 8c, d). The parenchyma cells were highly vacuolated and non-glandular. Intercellular spaces occurred between the parenchyma cells. Six collateral vascular bundles were found in the parenchyma. Groups of glandular trichomes occurred between the places where vascular bundles were localised (Fig. 8c), but they were sometimes found above the vascular bundle (Fig. 8e). The sessile glandular spur trichomes were composed of a single basal cell, a unicellular short pedestal cell (barrier cell with a thick radial wall impregnated with cutin) and a multi-celled head. The head cells were transfer cells (not shown). The inner spur epidermis formed conical papillae with cuticular striations (Fig. 8f).

**Fig. 9:**
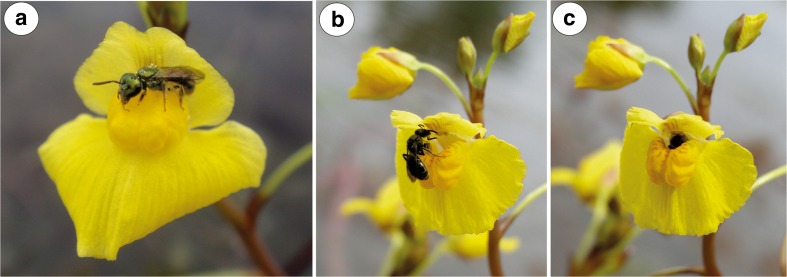
Pollination of *Utricularia foliosa*. **a**–**c** Behaviour of bees on *U. foliosa* flowers; note pollen grains on the bee thorax. (Credit of images: Dasmiliá Cruz)

#### Floral visitors and pollinators

*Utricularia foliosa* flowers were visited by two species of small Halictidae bees (Fig. 9a–c) which penetrated the palate and the corolla tube (Fig. 9b). *Utricularia* pollen grains were visible on the surface of the bee thorax (Fig. 9a).

## Discussion

Spur trichomes of *Utricularia* have been analysed for a long time—mainly for the potential taxonomic use (e.g. Farooq 1963; Farooq and Siddiqui 1966; Thor 1988; Taylor 1989). To date, however, no data have been published on the ultrastructure of spur nectary trichomes of *Utricularia*. All species examined here had the capitate trichome type with the single-barrier cell in the spur. The interspecific differences included the stalk length (these trichomes were sessile in *U. foliosa*, in *U. bremii* nearly sessile in contrast to well-stalked trichomes in *U. vulgaris* and *U. australis*) and the occurrence of the additional cell or cells in the stalk (*U. vulgaris*, *U. australis*). Płachno et al. (2017b) suggested that the general structure of the nectar-secreting trichomes in the genus *Utricularia* were very conservative in evolutionary terms, and results obtained here also corroborate this hypothesis. In all examined species in this study, the spur epidermis completely lacked nectary stomata and papillae were not glandular, either. Thus, the only source of nectar is the glandular capitate trichomes. Trichome head cells have ultrastructure typical for nectary glandular cells (see e.g. Fahn 1979a, b; Durkee 1983; Razem and Davis 1999; Wist and Davis 2006; Nepi 2007). However, it should be underlined that the most published observations were performed on the non-trichome nectarines, and data about trichome nectary ultrastructure are scarce and based on a few taxa only (*Lonicera*—Fahn and Rachmilevitz 1970; *Abutilon*—Robards and Stark 1988; *Hibiscus*—Sawidis et al. 1987, 1989; *Platanthera*—Stpiczyńska et al. 2005; *Cyclanthera*—Nepi 2007; *Adenocalymma*—Gama et al. 2016). In nectar-secreting trichromes, e.g. in *Abutilon* or *Hibiscus*, granulocrine secretion type was suggested (Kronestedt-Robards et al. 1986; Sawidis et al. 1987, 1989; Robards and Stark 1988; Kronestedt-Robards and Robards 1991). In head cells, we observed neither a “secretory reticulum” nor a hypertrophied Golgi apparatus. Moreover, the occurrence of cell wall ingrowths in the head cells may be evidence that the nectar is transported via an eccrinous mode of secretion (see Lüttge 1971; Nepi 2007).

The ultrastructural characters of the examined trichomes are very similar when compared with the scent glands from the palate of *U. bremii* (Płachno et al. 2017a) and the corolla appendages of *U. dunlopii* (Płachno et al. 2016). In all *Utricularia* species examined here, the nectary trichome had a barrier cell (pedestal cell) with a thick radial wall, which was impregnated with a lipophilic material. The endoderm-like element is typical for extranuptial nectaries devoid of stomata (see Paiva 2017 and literature therein). According to Cardoso-Gustavson and Davis (2015), cells impregnated with lipophilic compounds in the anticlinal walls not only act as a barrier to reduce an apoplastic route of outflow, but also control the inflow from the extracellular environment into actively secreting nectary tissues in floral trichome nectaries. However, it should be added that the barrier cell is the common character of various secretory trichomes of Lentibulariaceae (e.g. Fineran and Lee 1975; Fineran and Gilbertson 1980; Fineran 1985; Płachno et al. 2007, 2017a).

Based on our anatomical and ultrastructural observations and recent knowledge of the functioning of the nectaries (for details see Nepi 2007), we propose a way of nectar formation and secretion in *Utricularia*. Carbohydrates produced in tissues other than the spur are transported via vascular bundles to the spur parenchyma and epidermis cells and stored as starch. After starch hydrolysis, this pre-nectar is transported via the apoplast and the symplast from parenchyma and epidermis cells to the basal cell of the trichome. If there is an additional cell, this cell mediates the transport between the basal and barrier cells. The pre-nectar is transported between the basal and pedestal cell only via symplast as the radial wall of the barrier cell is impregnated and acts as a hydrophobic barrier. Also, the pre-nectar has to be transported only via symplast from the pedestal cell to the head cells. The pre-nectar is then transformed into nectar in the head cells. As these cells are transfer cells, the nectar secretion should be eccrine. Then, the nectar is accumulated in the subcuticular space and released via cuticle pores.

Even though the spur of *U. bremii* had a different size and shape (see Taylor 1989) from the other examined species of the same *Utricularia* section, the general anatomy of the spur was the same. However, there were some differences between species in the internal spur epidermis microsculpture, which may relate to specialisation for insect visitors/pollinators. All examined species were different from each other in the nectary trichome distribution at the spur surface: *U. vulgaris* (on the internal abaxial side of the spur), *U. australis* (on both the abaxial and adaxial side), *U. bremii* (on the internal abaxial side) and *U. foliosa* (in two oblong patches; Thor 1988; Taylor 1989 and our observations). The close localisation of the nectar trichomes near the vascular bundles in *U. foliosa* is probably due to shortening the route of sugar transport from the phloem to the nectary tissue or to an increased water demand for the nectar production. A similar connection between nectar trichome localisation and vascular tissue was recorded in the *U. nelumbifolia* spur, where the trichome distribution in some parts of the spur coincided with the position of the vascular bundles (Płachno et al. 2017b: Fig. 12A). According to Silva et al. (2018), the generic section *Utricularia* is subdivided into two major clades, one formed by *U. floridana*, *U. gibba* and *U. striata* (clade I) and the other one (clade II) by the remaining species including those analysed here. However, *U. foliosa* is phylogenetically the most distant species within clade II, nesting outside the core of the section *Utricularia. Utricularia vulgaris* is most related with *U. australis*, and this fact is also reflected in the nectar trichome structure.

We found that the examined species differed in the micromorphology of papillae in the spurs. Papillae with striations occurred in *U. bremii* and *U. foliosa*, whereas only smooth papillae were observed in *U. australis* and *U. vulgaris*. Also, *U. bremii* had papillae with rounded tips in contrast to other species. Unfortunately, there is only scarce literature information on the micromorphology of papillae in *Utricularia* spurs; papillae with striations were recorded in *U. dunlopii* and *U. dichotoma* (section *Pleiochasia*; Płachno et al. 2016), *U. reniformis* (Clivati et al. 2014), *U. nelumbifolia* and *U. cornigera* (Płachno et al. 2017b; formerly sect. *Iperua*, now included in sect. *Orchidioides*, see Rodrigues et al. 2017). Thus, it is difficult to draw any evolutionary conclusions. Various papillae sizes and surface morphology (with smooth surface versus papillae with striations) may provide “tactile information” for particular pollinators. According to Kevan and Lane (1985), honeybees are able to detect, learn and discriminate between the microsculptured epidermis of flower petals. The microsculptural patterns can be used as nectar guides by foraging insects. Bell et al. (2009) proposed that the occurrence of papillae in spurs of deceit-pollinated orchids may improve pollination because papillae satisfy the tactile expectation of pollinating insects. The occurrence of papillae in *Utricularia* spurs may also have another explanation. Papillae form a hydrophobic surface and so, plants may produce less nectar. This explanation is in an agreement with the observation of Hobbhahn et al. (2006) who recorded extremely small volumes of nectar in spurs of some terrestrial *Utricularia* species. These authors also observed that nectar did not accumulate in the tip of the spur but adhered in small droplets to the inner spur walls. We have made a similar observation in *U. vulgaris*. Thus, these micro-droplets are formed on a single trichome head or a group of trichomes. However, Clivati et al. (2014) observed that in the *U. reniformis* spur, nectar occurred as droplets but it also reached down to the spur tip.

Taylor (1989) mentioned Hymenoptera, Diptera, Lepidoptera and hummingbirds as visitors to *Utricularia* flowers. Hobbhahn et al. (2006) observed that more than 50 species of bees, butterflies, moths, hawk moths and dipterans visited and pollinated the flowers of three terrestrial *Utricularia* species belonging to the subgenus *Bivalvaria*.

Even though *Utricularia vulgaris* is not only far-ranging but also one out of the best known *Utricularia* species, the literature is lacking both pollination biology studies and reports of pollinator observations. In general, observations of pollinators or flower visitors of *Utricularia* species from the section *Utricularia* are scarce. Araki and Kadono (2003) mentioned that Japanese *Utricularia australis* (probably this was *U. tenuicaulis* Miki) was pollinated “by small insects, such as aphids or small dipterous species”. However, these authors did not add any details or documentations, which might prove that these insects were pollinators. An aphid *Rhopalosiphum nymphaeae* was recorded as a pest on *Utricularia* spp. by various authors (see e.g. Center et al. 1999), but we have observed that aphids mainly occupy external parts of inflorescence. Honda (2007) observed that flowers of *Utricularia macrorhiza* (closely related to *U. vulgaris*) were visited and pollinated by a member of the Syrphidae family, which was determined as *Helophilus intentus* (see http://pollinator.org/shop/poster-15). *Helophilus* behaviour on the *U. macrorhiza* flower reminds that instead one of *Eristalis tenax* on *U. vulgaris* flowers. Westerkamp and Classen-Bockhoff (2007) and later also Clivati et al. (2014) classified *Utricularia* as having gullet-shaped flowers. In this type of flower, reproductive structures are located in the upper side of the corolla and, consequently, the pollen grains become attached to the pollinator’s back—nototribic transfer (Faegri and van der Pijl 1971). We documented the nototribic transfer in *U. vulgaris* because we found *Utricularia* pollen grains at the *Eristalis* back. However, pollen grains were also found at other insect parts (e.g. near eyes) and this is due to that the fly cleaned its body surface after penetrating the flower.

Salmon (2001) mentioned small honey bees as pollinators of *Utricularia gibba* L., however, without any details on the pollination biology. Płachno et al. (2017a) observed that small Hymenoptera (members of families Mymaridae and Braconidae) were flower visitors of *U. bremii*. They suggested that these Hymenoptera might also be pollinators of *U. minor*, which has a similar size and flower structure to those of *U. bremii*.

Both *U. vulgaris* and *U. foliosa* flowers were visited by small Halictidae bees. As we did not observe *U. vulgaris* pollen grains on the surface of the *Lasioglossum* bee head and thorax, we propose that this bee is only a flower visitor in contrast with the true pollinator *Eristalis tenax* fly. However, in *U. foliosa*, the small Halictidae bees seem to be pollinators of this species.

## Conclusions

Nectar in *Utricularia* flowers is produced by capitate spur trichomes and despite the various spur morphology, the trichomes have similar architecture and ultrastructure. The head cells of these trichomes are transfer cells with an eccrine nectar secretion. As the flower spur in *Utricularia* is the organ where nectar is produced and stored, it should be considered nectary.
